# Malaria and tuberculosis co-infection—a review

**DOI:** 10.1093/oxfimm/iqad008

**Published:** 2023-11-15

**Authors:** Else M Bijker, Sanjay Deshpande, Padmini Salgame, Rinn Song

**Affiliations:** Department of Paediatrics, Oxford Vaccine Group, University of Oxford, Oxford, OX3 7LE, UK; Department of Paediatrics, Maastricht University Medical Center, MosaKids Children’s Hospital, Maastricht, 6229 HX, The Netherlands; Department of Paediatrics, Rainbow Children’s Hospital, Hyderabad, Telangana, 500 034, India; Department of Paediatrics, Oxford Vaccine Group, University of Oxford, Oxford, OX3 7LE, UK

**Keywords:** malaria, tuberculosis, immune response, immunology

## Abstract

Malaria and tuberculosis remain highly prevalent infectious diseases and continue to cause significant burden worldwide. Endemic regions largely overlap, and co-infections are expected to occur frequently. Surprisingly, malaria-tuberculosis co-infection is relatively understudied. Malaria has long been known to have immunomodulatory effects, for example resulting in reduced vaccination responses against some pathogens, and it is conceivable that this also plays a role if co-infection occurs. Data from animal studies indeed suggest clinically important effects of malaria-tuberculosis co-infection on the immune responses with potential consequences for the pathophysiology and clinical course of both infections. Specifically, rodent studies consistently show reduced control of mycobacteria during malaria infection. Although the underlying immunological mechanisms largely remain unclear, an altered balance between pro- and anti-inflammatory responses may play a role. Some observations in humans also support the hypothesis that malaria infection skews the immune responses against tuberculosis, but data are limited. Further research is needed to unravel the underlying immunological mechanisms and delineate possible implications of malaria-tuberculosis co-infection for clinical practice.

## Introduction

Malaria and tuberculosis (TB) remain among the leading infectious diseases causing morbidity and mortality worldwide. About one-quarter of the world’s population is infected with TB, and in 2021 an estimated 10.6 million people developed TB disease, with approximately 1.6 million deaths [1]. In the same year, malaria was estimated to cause more than 240 million cases and 619 000 deaths. The COVID-19 pandemic has disrupted health services, reversing years of progress in disease control [2–5]. Moreover, increasing drug resistance complicates clinical management and public health efforts for both infections [6, 7]. Both malaria and TB are endemic in large areas of the world, and it can be assumed that co-infections frequently occur in high-transmission regions. While there is a considerable body of literature on malaria/HIV and TB/HIV co-infection, much less attention has been given to clinical and immunological aspects of malaria/TB co-infection [8]. This review aims to provide an overview of the existing literature emphasizing immunological aspects of malaria/TB co-infection.

Malaria has a complex life cycle. The protozoan *Plasmodium* parasites are transmitted to humans through the bites of infected female *Anopheles* mosquitoes. The injected sporozoites move to the liver, invade hepatocytes, and mature and multiply. Once the infected hepatocytes burst open, merosomes containing merozoites are released into the bloodstream with exponentially increasing parasitaemia due to repeated invasion and multiplication in erythrocytes. Symptoms may occur as soon as parasites occur in the blood [9]. If not treated promptly and adequately, *P. falciparum* infection may result in severe anaemia, cerebral malaria, acute respiratory distress syndrome, organ failure, or death in non-immune individuals [9]. Some blood-stage parasites develop into gametocytes, which cause infection of a mosquito when it takes a blood meal from the human host. Subsequently, sporozoites develop in the salivary glands of the infected mosquito, completing the life cycle of the *Plasmodium* parasite [10].

*Mycobacterium tuberculosis* (MTB) bacilli are transmitted through aerosols from an infected individual to another person. In most immunocompetent individuals, the mycobacteria are phagocytosed by macrophages and contained after inhalation, resulting in latent infection [11]. Acute primary infection occurs in a small proportion, more commonly in young children and immunocompromised hosts. Haematogenic or lymphatic spread can result in severe disease such as miliary TB or meningitis [12, 13]. About 5–10% of individuals with latent TB will develop active TB disease later in life [14].

## Epidemiology

TB and malaria are ancient infections, with co-infections demonstrated using molecular techniques in mummified remains from lower Egypt dating back to circa 800 BC [15]. Some authors have suggested that this might have resulted in a mutually beneficial co-evolvement [8, 16], but this hypothesis is hard to substantiate.

It is difficult to ascertain the prevalence of co-infections, with a considerable variation in reported rates. In a cross-sectional study in the early 2000s in Mwanza, Tanzania, newly diagnosed patients with TB were screened for co-infections. Out of 655 patients with TB, 4.1% had malaria parasites in their blood. This did not differ significantly between the microbiologically confirmed and clinically diagnosed TB groups, nor did the geometric mean parasitaemia, with 378 and 928 parasites/µl, respectively [17]. In a similar cross-sectional study in Kampala, Uganda, eight out of 363 patients with TB (2.2%) had *P. falciparum* parasitaemia [18]. In Limbe, Cameroon, of 400 participants, 1.5% had malaria/TB co-infection, while TB mono-infection occurred in 11.8% and malaria in 23.3% [19]. Co-infection was much more common in a retrospective study from Luanda, Angola, which showed that 37% of all patients with TB had malaria parasitaemia [20]. An ecological study using epidemiological surveillance data from the Brazilian Amazon demonstrated a spatial association between malaria and TB infections, independent of socio-economic factors [21]. The large variation in reported co-infection rates could be explained by actual epidemiological differences and variations in malaria transmission patterns but is also influenced by available diagnostic techniques and case definitions. Moreover, the potential consequences of these co-infections are challenging to interpret due to the lack of appropriate control groups in these studies.

## Clinical aspects of malaria/TB co-infections

It is conceivable that malaria/TB co-infection is a common scenario in endemic regions, but there is limited data on its clinical implications. The clinical presentation of malaria varies with age in endemic areas. Young children often experience severe symptoms, including high fever and anaemia, and are at a higher risk of mortality. In contrast, older individuals may exhibit asymptomatic parasitaemia, due to acquired immunity over time.

A study in Guinea-Bissau demonstrated that malaria prevention through bed nets, cotrimoxazole prophylaxis during the wet season and insecticide spraying resulted in a reduction of mortality from 26 to 19% in patients admitted with TB [22]. Even though the interpretation of these results is limited because of the lack of a control group and the potential effect of cotrimoxazole on *M. tuberculosis*, it supports the notion that malaria co-infection could be a clinically important phenomenon.

A study in Cameroon showed a trend toward lower haemoglobin (Hb) levels in malaria/TB co-infected patients compared to mono-infections. While the average Hb was 13.4 g/dl (SD 2.6) for patients with TB and 11.4 g/dl (SD 2.3) for malaria, the average Hb was lower with 10.4 g/dl (SD 2.6) in co-infected patients [19]. This study did not report the severity of the malaria infection, and the age range in this study was 15–49 years with a mean age of 33 years, an age at which most parasitaemia is asymptomatic in countries with high transmission [23].

Another study showed a reduction in total white blood cell count, neutrophils and lymphocytes in patients with malaria/TB co-infection compared to TB mono-infection [24], although the clinical significance of this observation is unclear. A case report from India described concurrent infection with *Plasmodium falciparum* and TB in a 34-day-old child, indicating that clinicians should be aware of this possibility in all age groups [25].

An important clinical consideration when treating co-infections involves the side effects and potential drug interactions. Since endemic areas largely overlap and both diseases are highly prevalent, concomitant treatment may occur. One important issue is QT-prolongation in the case of fluoroquinolones and many anti-malarials [26]. Fluoroquinolone-based TB treatment regimens have become increasingly important. The use of many anti-malarials, including artemether/lumefantrine and chloroquine in combination with fluoroquinolones is currently contra-indicated, and clinical studies are needed to evaluate the risk. Another drug-interaction to consider in treatment co-infections is that rifampicin induces the hepatic enzyme cytochrome P450, reducing serum concentrations of many drugs, including artemisinin, lumefantrine, atovaquone and mefloquine [27]. This increases the risk of recrudescent infection, and patients should be monitored closely [27].

## Immunology

### A brief summary of immunity against malaria

Naturally-acquired immunity against malaria develops gradually after repeated exposure occurring over the years. This is evidenced by the observation that young children suffer the largest disease burden in endemic countries. Antibodies that prevent the sequestration of infected erythrocytes and inhibit the inflammatory cytokine response probably play an important role here. With increasing age, presumably due to repeated infections, children and adults become resistant to severe disease [28]. On a population level, disease becomes milder at increasing age, with eventually asymptomatic carriage in those individuals who have become resistant to disease while still susceptible to re-infections [28]. Ongoing exposure is required to maintain immunity: immune individuals who relocate to a non-endemic area are thought to regain susceptibility [29]. While naturally-acquired immunity is mainly directed against the blood stages of the parasite, experimental immunization and vaccination against the pre-erythrocytic stages can induce sterilizing immunity [30, 31].

Immunity to malaria is complex, inherent to the different intra- and extracellular lifecycle stages of the parasite. Innate responses against *Plasmodium* have not been elucidated entirely yet but include splenic removal of infected erythrocytes, opsonin-independent phagocytosis by macrophages and interferon-gamma (IFN-γ) production by NK cells [32]. Moreover, innate immune cells such as dendritic cells and NK cells have an important role in modulating the adaptive immune response through the production of cytokines [32].

Adaptive immunity develops gradually due to repeated infections and involves both humoral and cellular immunity. Antibodies are important in the protection against malaria, as demonstrated elegantly in the 1960s through the adoptive transfer of serum from immune to naïve individuals [33]. Antibodies protect against malaria through several different mechanisms: they block the invasion of sporozoites into liver cells and the invasion of merozoites into erythrocytes [34].

Although much is still unclear about the role of various T-cell subsets in anti-malaria immunity, they appear to play a critical role against both pre-erythrocytic (sporozoites/liver stages) and erythrocytic parasites [35]. In animals and probably also humans, IFN-γ and CD8+ T cells contribute to pre-erythrocytic immunity by abrogating parasite development in hepatocytes. CD4+ helper T cells orchestrate the parasite-specific antibody responses against both sporozoites and blood stages. Macrophages phagocytose merozoites and infected red blood cells, activated and directed by CD4+ T cells [29].

There is also a role for the host immune response in malaria pathology, as demonstrated by the correlation between the severity of the disease and levels of pro-inflammatory cytokines such as TNF-α [36, 37]. Indeed, in rodent models, the absence of IFN-γ of IL-2 alleviates pathology [38].

### A brief summary of immunity against TB

The immune response to MTB varies according to age and involves both innate and adaptive immune system components. Macrophages, neutrophils, natural killer cells, and dendritic cells, along with airway epithelial cells, are the first to respond to the inhaled tubercle bacilli [39]. Once mycobacteria are inhaled into the terminal alveoli, they are readily phagocytosed by alveolar macrophages and dendritic cells that process and present the antigens and migrate to regional lymph nodes where they present MTB antigens to T cells. An array of cytokines, including Interleukins (IL), IL-12, IL-23, IL-6, IL-1, tumour-necrosis factor-alpha (TNF-α) and IFN-γ are released during infection and drive the immune response and containment of the infection [40]. The importance of cellular immunity in controlling MTB infection is demonstrated by increased susceptibility to infection and severity of TB disease in individuals with low CD4+ T cells, for example, in HIV infection [41]. The importance of the IFN-γ/IL-12 pathway is illustrated by the pathophysiology of Mendelian susceptibilities to mycobacterial disease (MSMD), with inborn errors leading to increased susceptibility and sometimes disseminated, life-threatening mycobacterial infection [42].

TNF-α facilitates macrophages to phagocytose and kill mycobacteria and is important in containing infection, reflected by the increased risk of TB in individuals receiving anti-TNF-α treatments [43]. Granulomas are the hallmark of mycobacterial infection; immune effector cells such as macrophages, T- and B-lymphocytes form a structured unit surrounding a central core where viable mycobacteria can persist while their replication is limited [44].

About one-quarter to a third of the world’s population is estimated to be infected with *M.* tuberculosis [1]. In approximately 90–95% of these latently infected individuals, the stand-off between the host immune response and bacterial replication continues lifelong. In the remaining 5–10% of the infected population, active disease develops at some point in life, with reactivation of bacterial replication and disruption of the granuloma. Mechanisms underlying reactivation are incompletely understood, but animal models demonstrate a role for reduced CD4 T cell function [45]. The increased number of TB cases in individuals on TNF-α blocking medication suggests an important role for this cytokine [43, 46].

## Immuno-modulatory effects of malaria infection

Malaria has long been known to have immunomodulatory effects. This is illustrated by observational data on vaccination responses and viral, bacterial and parasitic co-infections. In the 1970s, it was shown that if children with acute malaria infection were vaccinated against meningococcus or *Salmonella typhi*, they generated significantly fewer specific antibodies than healthy controls [47]. Even if children were vaccinated a month after the acute malaria infection, the response to the meningococcal vaccine was still impaired [48].

The presumed role of Epstein Barr Virus (EBV) and malaria co-infection in the pathogenesis of Burkitt lymphoma prompted a study that demonstrated significantly higher EBV loads in young children in a region with holoendemic malaria transmission compared to children of the same age from an area with sporadic malaria transmission [49]. Although the underlying mechanism remains unclear, it seems reasonable to assume that the immunomodulatory effects of malaria infection affected the EBV viral load. In Gambian children, the proportion of children with circulating Hepatitis B surface antigen was significantly higher amongst children with severe malaria than in children with mild malaria or without malaria infection [50]. While this could result from malaria-induced immunosuppression, it could also be the opposite, where active hepatitis B infection predisposes to severe malaria, or there could be another common risk factor for both diseases.

An association between non-typhoid salmonella septicaemia and recent malaria infection was found in children from the Gambia [51]. Moreover, PCR analysis of blood samples from febrile children in Gabon showed that bacterial DNA was more frequently found in children who also had *Plasmodium* in their blood than in those who had not [52].

In mice, the expulsion of the nematode *Trichuris muris* is suppressed if they are simultaneously infected with the murine malaria parasite *Plasmodium berghei* [53].

Immunization of healthy subjects with viable *Plasmodium falciparum* sporozoites while on chloroquine prophylaxis can induce sterilizing protection against subsequent controlled challenge [54]. However, when immunization is performed in the presence of blood-stage parasitaemia, this protection is abrogated, suggesting a negative effect of blood-stage parasites on the induction of pre-erythrocytic immunity [55].

## Malaria/TB co-infection

### Animal studies

Animal models have contributed significantly to understanding the immunological effects of malaria/TB co-infection, consistently showing reduced control of MTB during malaria infection. Hawkes *et al*. investigated co-infection dynamics in a non-lethal C57BL/6 mouse model of disseminated *M. bovis* BCG and *Plasmodium chabaudi chabaudi* AS [56]. A higher mycobacterial load was observed in the spleens and livers of mice concomitantly infected with malaria compared to mice with BCG infection alone. Moreover, disease severity was more pronounced in co-infected mice, as reflected by weight loss and splenomegaly. The underlying immunological mechanisms remained unclear, as blood TNF and IFN-γ levels did not differ between co-infected and control animals. In an *in vitro* follow-up experiment, MTB proliferated in macrophages approximately two-fold higher when co-cultivated with *P. falciparum-*infected red blood cells compared to uninfected red blood cells [56].

Mueller *et al*. established a rodent model of MTB and *P. berghei* co-infection [57]. They challenged C57Bl/6 mice with an established pulmonary MTB infection with *P. berghei NK65-infected* mosquito bites. MTB load in the lungs of co-infected mice was significantly higher after 12 days compared to control mice. Moreover, co-infected mice had more severe lung pathology with increased inflammation [58]. Also, Scott *et al*., who used a *P. yoellii-*MTB co-infection model, showed that co-infected mice had increased MTB load in their lungs, spleen and liver and had increased mortality, with a concurrent increased number of IFN-γ producing CD4 T cells [59]. In another mouse model using *P. yoelii,* these findings were confirmed. When sacrificed after fifty-one days, co-infected mice showed a slight increase in MTB burden in the lungs, spleen, and liver, suggesting that malaria infection limited the control of MTB. It has been difficult to disentangle the underlying immunological factors, but it probably involves an altered balance between pro- and anti-inflammatory responses since co-infected mice showed an increased number of immune cells and levels of both pro- and anti-inflammatory cytokines such as IFN-γ and IL-10 [60]. Moreover, *Plasmodium* appears to influence granuloma formation, delaying this process in a *P. yoelii-*BCG co-infection model in BALB/c mice [61].

Reduced CD4+ T cell counts and TNF-α blockage can trigger TB reactivation, but much remains unknown. Interestingly, in mice with latent BCG infection, malaria infection resulted in the reactivation of mycobacterial infection with inflammation in the spleen and liver, a rise in mycobacterial counts and disorganization of granulomas [56].

The effect of malaria on BCG-induced protection against TB appears to be limited. In a C56BL/6 mouse model, neither *Plasmodium berghei* nor *Plasmodium chabaudi* infection abrogated BCG-induced protection against TB, even though the malaria infections significantly affected both B- and T-cell immunity such as central memory T lymphocyte apoptosis and reduction of marginal zone and follicular B-cell populations [62]. Moreover, protection against MTB challenge by four different TB vaccines, including BCG, was not impaired by *P. yoelii* infection in C57BL/6 mice in another study [63].

The effect of TB co-infection on the course and pathophysiology of malaria is less clear. Co-infection of C57BL/6 mice with *M. tuberculosis* and *Plasmodium yoelii* 17XL resulted in a lower peak parasitaemia and increased survival compared to *P. yoelii* 17XL mono-infection [64]. Enhanced expression of pro-inflammatory genes, including IFN-γ, TNF-a, STAT 1, and other IFN-γ-inducible genes in co-infected mice suggests that this might play a role in the relative protection. However, the augmentation of IFN-γ responses was not observed in BALB/c mice, and MTB co-infection did not result in protection from lethal malaria [64]. In another study, previous infection with MTB did not alter the clinical course of experimental cerebral malaria caused by *P. berghei* ANKA in C57BL/6 mice, nor were there any significant alterations in cytokine and T-cell responses [65]. These observations emphasize the importance of differences between hosts and parasite strains and the need for caution when translating animal data to humans.

### Human studies

In humans, data on immunological aspects of malaria/TB co-infection are limited. A cross-sectional study from Nigeria showed a significant reduction of IL-10 and IL-6 in co-infected patients compared to TB mono-infection. TFN-α levels were not different, and IFN-γ was not measured [66]. The authors did not provide data on the potential clinical consequences of these differences.

Some *ex vivo* data, however, suggest an effect of malaria on TB-specific immune responses. For example, the lymphoproliferative response to purified protein derivate (PPD) was lower in the majority of healthy adults during the transmission (wet) season compared to the dry season in a study in Sudan [67]. Why this was the case is unclear since the difference remained when individuals with parasitaemia were excluded. In another study, serum from *Plasmodium falciparum*-infected individuals suppressed the *in vitro* lymphoproliferative response to *Plasmodium* antigens and PPD [68]. This was supported by a study in Sudan, where patients with uncomplicated malaria showed reduced lymphoproliferative responses to PPD during the acute phase of the disease, which recovered during convalescence [69].

An evaluation of data from refugee health assessments in Perth, Australia, showed that having malaria was associated with a three-times higher chance of having an indeterminate QuantiFERON-TB Gold IFN-γ release assay (IGRA) result [70]. This was further specified in a study in Tanzania, which demonstrated that individuals with malaria parasites in their blood had higher IFN-γ levels in the unstimulated samples and lower IFN-γ levels in the mitogen tube [71]. Unfortunately, it was impossible to draw conclusions regarding the MTB-specific results due to sample size limitations. In a household contact study of sputum-positive patients with TB in Uganda, there was no association between malaria co-infection and QuantiFERON-TB Gold In-Tube positivity at baseline or six months follow-up [72]. Unfortunately, data were inadequate to assess the potential effect of malaria on the reactivation of TB disease.

Stimulation of PBMCs isolated from Nigerian adolescents and adults with PPD showed similar IFN-γ production but increased IL-4 and IL-10 secretion in malaria/TB co-infected subjects compared to individuals with TB mono-infection [73]. This finding supports the animal dating suggesting that malaria skews the immune against TB towards a more Th2/anti-inflammatory response with potential consequences for bacterial control and disease progression, given the importance of Th1 responses in the effector response against mycobacteria.

Results on the effect of placental malaria on PPD-specific responses are conflicting. A study in Gambian infants suggested a reduced proportion of PPD-specific IFN-γ producing CD4+ T cells in infants with histological evidence of placental malaria compared to healthy controls [74]. In another study, however, neither proliferative responses nor IFN-γ production of cord blood mononuclear cells to PPD significantly differed when isolated from placentas with or without *Plasmodium* parasites [75].

Regulatory T (Treg) cells are a subset of CD4+ T cells defined by expression of the transcription factor FOXP3 and have been recognized to have an important role in regulating immune homeostasis [76]. By limiting excessive inflammation, they reduce the damaging effect of the immune system on the host. But on the other hand, this can result in less effective pathogen clearance and memory responses [77]. In *Plasmodium* blood stage infection, Treg cell populations expand, and their frequencies are associated with improved outcomes and lower parasitaemia [78]. Due to the inherent limitations of immunological studies in humans, it remains unclear what are cause and effect, with varying reports from field studies [77, 79]. Similarly, studies of peripheral blood show increased numbers of Tregs in patients with active TB disease compared to those with latent infection or healthy controls [80]. And again, it is unclear whether these Tregs are the result of the infection or a risk factor for developing TB disease. As both infections induce Tregs, cross-modulation is a plausible mechanism for some of the phenomena seen in co-infection, and the delineation of this interaction deserves further study.

## Effects of BCG vaccination on malaria

There is ample experimental evidence for the relative protection of BCG vaccination against malaria, most likely through a non-specific modulation of the immune response. Animal studies from the 1970s demonstrated a nonspecific protective effect of BCG vaccination against *Babesia microti* and *P.berghei yoelii 17X* malaria [81]. This was reproduced in other studies, showing that BCG vaccination protected B6D2 F1 mice from lethal *P. berghei* challenge [82]. A follow-up study confirmed the protective effect of BCG on *P. yoelii 17XL* challenge in A/J mice concurrent with a shift towards Th1 response with higher levels of IFN-γ and lower levels of IL-4 in BCG-vaccinated mice compared to controls and enhanced the production of *Plasmodium*-specific IgG2a antibodies. Neutralization of IFN-γ abrogated protection [83]. In another, non-lethal C57Bl/6 *P. yoelii* rodent model, parasitaemia was significantly lower in BCG-vaccinated animals. Subsequent depletion experiments showed a critical role for both CD4+ and CD8+ T cells [84].

BCG also protected C57Bl/6 mice from *P. berghei ANKA-*induced experiment cerebral malaria [85]. Interestingly, this was not associated with a reduced parasite load but with the reduction of pro-inflammatory cytokines, chemokines, and CD8+ T-cells in the brain of BCG-vaccinated animals. However, the protective effect of BCG was not evident for all rodent *Plasmodium* species. C57Bl/6 mice had higher levels of parasitaemia and increased mortality from *P. c. chabaudi AS* infection if they had received BCG four weeks earlier, associated with an increased proportion of CD4 + CD25 + Foxp3 + regulatory T cells and reduced levels of IFN-γ and TNF-α [86].

Some observational data support the hypothesis that BCG induces non-specific protection against malaria in humans. For example, in Guinea-Bissau, analysis of mortality patterns showed that having a BCG scar significantly reduced the risk of death from malaria [87]. Similarly, a study from the same country evaluated data from three randomized controlled BCG trials with >6500 infants and showed that the non-specific effect of BCG on reducing all-cause mortality was most prominent when administered during the malaria season [88]. Moreover, in a large multinational study using demographic health survey data from more than 34 000 children from 13 sub-Saharan countries, BCG vaccination was associated with a small but significant reduction in malaria prevalence [89]. BCG vaccination did not protect against *P. falciparum* in a controlled human malaria infection model, although BCG-vaccinated individuals developed earlier symptoms and more robust inflammatory responses, suggesting some extent of trained immunity [90].

## Conclusion

Malaria/TB co-infection is a surprisingly underexplored area, given the high prevalence of both infections and the large geographical overlap between endemic regions. A substantial amount of animal data suggests clinically important effects of co-infection on the immune response with potential consequences for diagnosis, pathophysiology and clinical course of both infections. Some observations in humans support these hypotheses, but data are limited, possible implications for clinical practice remain unclear, and many questions remain. Does malaria infection trigger the reactivation of TB in humans? Does malaria influence TB disease severity and vice versa? Knowledge evolution on both infections has suffered from a relative lack of funding compared to diseases with increased impact in high-income countries. Immunity to either infection is highly complex and incompletely understood, making it challenging to disentangle the crosstalk. Still, a systematic investigation of the pathophysiology of malaria/TB co-infection could provide important insights. This could include prospective studies evaluating the effects of co-infection and treatment on clinical and immunological outcomes, the effect of malaria on TB disease progression, and the interaction of infections with vaccination. Improved understanding may lead to more targeted interventions that could have a major impact on both diseases.

## Supplementary Material

iqad008_Supplementary_Data

## Data Availability

No new data were generated or analysed in support of this research.
