# Supplementary material for: Malaria and tuberculosis co-infection—a review
Source: Oxf Open Immunol. 2023 Nov 15;4(1):iqad008. doi: 10.1093/oxfimm/iqad008 (PMC10681873; doi:10.1093/oxfimm/iqad008)
Supplement: iqad008_Supplementary_Data [file iqad008_supplementary_data.pdf]

<u>Referee 1 Original Report – 008</u>

This is a really readable, novel review on an important topic.

While the actual paper delves into some fairly substantive discussion of immune mechanisms and interactions, the abstract is a rather generic overview and so gives the reader relatively little idea of the actual content. Would it be possible to get more of the immunology into the abstract?

Also, this paper would really benefit from a graphic.
